# Diagnosing left ventricular aneurysm from pseudo-aneurysm: a case report and a review in literature

**DOI:** 10.1186/1749-8090-4-11

**Published:** 2009-02-24

**Authors:** Giampaolo Zoffoli, Domenico Mangino, Andrea Venturini, Alberto Terrini, Angiolino Asta, Chiara Zanchettin, Elvio Polesel

**Affiliations:** 1 Department of Cardiac Surgery, Ospedale dell'Angelo, Via Paccagnella 11, 30174, Venice, Mestre, Italy

## Abstract

Rupture of the free wall of the left ventricle (LV) is a catastrophic complication occurring in 4% of patients after myocardial infarction (MI) and in 23% of those who die of MI. Rarely the rupture is contained by an adherent pericardium creating a pseudo-aneurysm. This clinical finding calls for emergency surgery. If no ruptures are detectable and myocardium wall integrity is confirmed, we are in the presence of a true aneurysm, which can be treated by means of elective surgery. Differentiation between these two pathologies remains difficult. We report the case of a patient with a true aneurysm, initially diagnosed as pseudo-aneurysm at our institution; we have reviewed the literature on this difficult diagnosis and outlined characteristic findings of each clinical entity.

## Introduction

Rupture of the free wall of the left ventricle (LV) is a catastrophic complication occurring in 4% of patients after myocardial infarction (MI) and in 23% of those who die of MI [1]. Rarely the rupture is contained by an adherent pericardium creating a pseudo-aneurysm. This clinical finding calls for emergency surgery. If no ruptures are detectable and myocardium wall integrity is confirmed, we are in the presence of a true aneurysm, which can be treated by means of elective surgery. Differentiation between these two pathologies remains difficult. We report the case of a patient with a true aneurysm, initially diagnosed as pseudo-aneurysm at our institution; we have reviewed the literature on this difficult diagnosis and outlined characteristic findings of each clinical entity.

## Case Report

B. A., a 71-year-old man, presented with dyspnoea under moderate exercise, without angina. His clinical history was characterized by a silent inferior-basal myocardial infarction detected by a control ECG that presented inferior Q waves, and he had not previously presented symptoms. The infarct was referable to maximum a couple of months before especially basing on symptoms worsening. So he had not any previous diagnosis or treatment of the acute event.

Functional NYHA Class was III, but it was I two months before, and symptoms were rapidly worsening in the last 20 days.

The patient was a smoker, affected by chronic obstructive pulmonary disease (COPD), hypertension, and peripheral vascular disease, presented in sinus rhythm. Medical therapy was based beta blockers, ASA, and a diuretic with some relief of symptoms.

**Figure 1:**
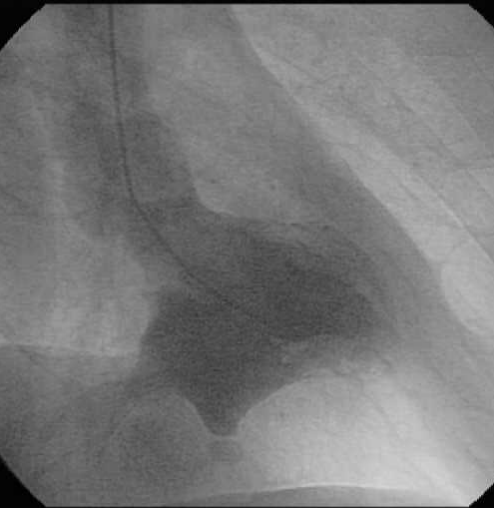
**Left ventricular angiography**. Left ventricular angiography showed an enlargement of the left ventricle with a large dyskinetic cavity localized in the diaphragmatic region, suggesting the presence of a pseudo-aneurysm.

A transthoracic echocardiogram (TTE) showed a dyskinetic posterior wall of the left ventricle, mild mitral regurgitation, with moderate reduction of the ejection fraction (EF = 44%). An aneurysmatic enlargement was noted in the posterior wall, but with few signs to distinguish a true aneurysm from a pseudo-aneurysm. The patient underwent a coronary angiographic examination that revealed patent anterior descending and circumflex arteries and a right coronary artery that was completely closed and perfused by hetero-coronaric circulation. Contrast ventriculography showed an enlargement of the left ventricle with a large dyskinetic cavity localized in the diaphragmatic region, suggesting the presence of a pseudo-aneurysm. (Fig. 1)

A second TTE was done, with results similar to the first one, but a thrombus in the posterior wall was also disclosed.

**Figure 2:**
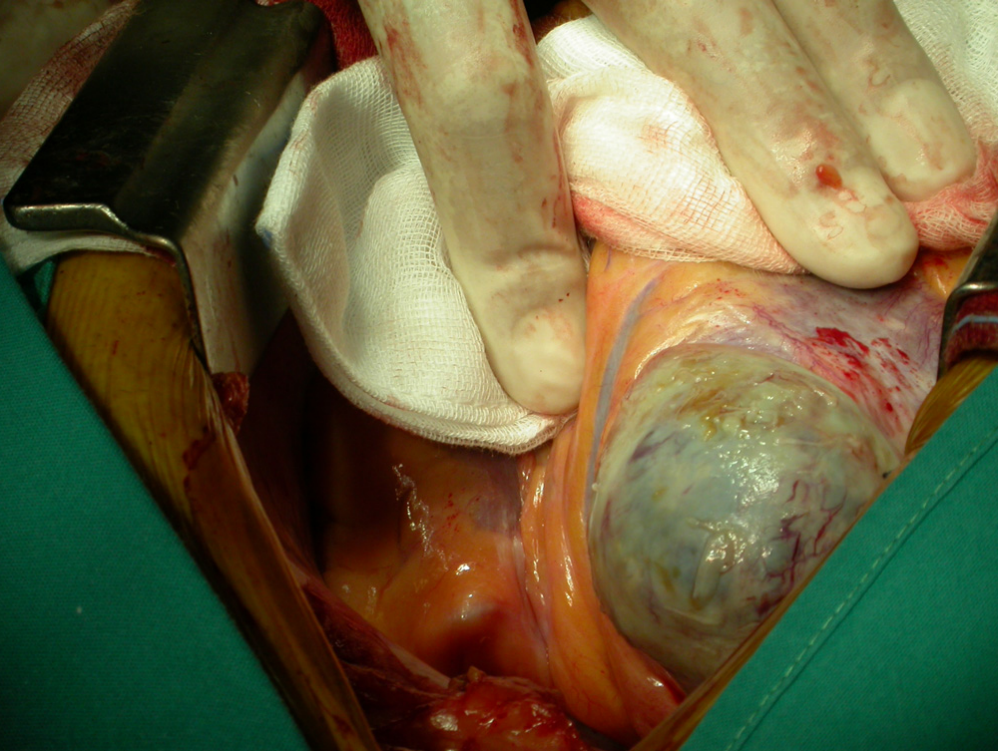
**Surgical time**. A large true aneurysm of the posterior wall of the left ventricle was found.

**Figure 3:**
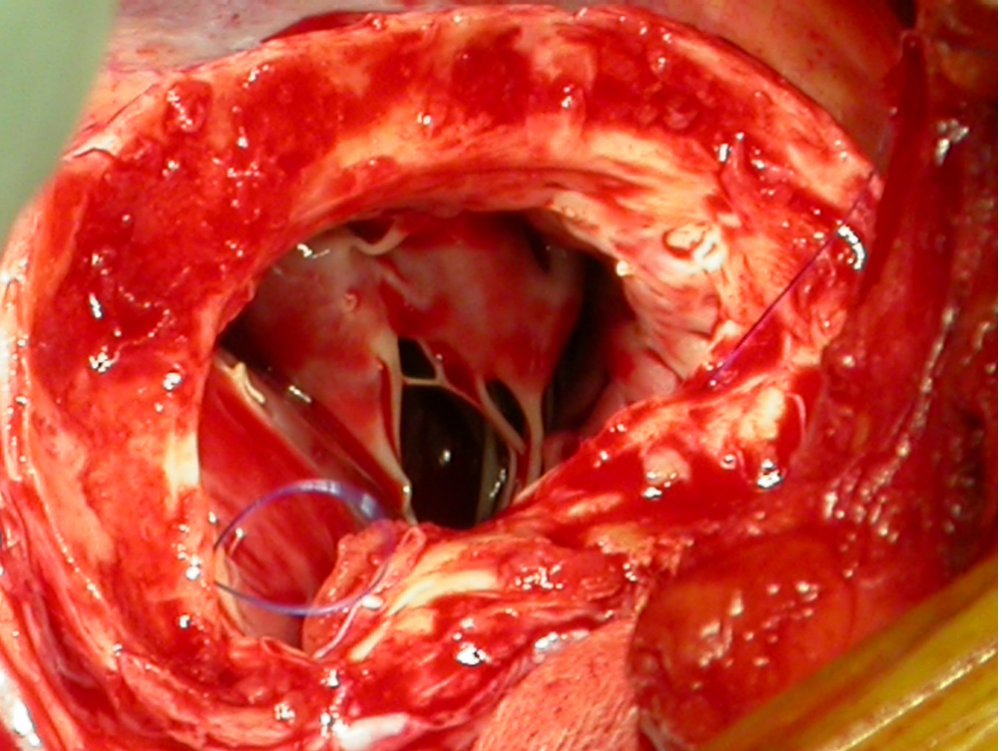
**The aneurysm was opened**. The aneurysm consisted of a very thin myocardium layer, close to the mitral valve.

**Figure 4:**
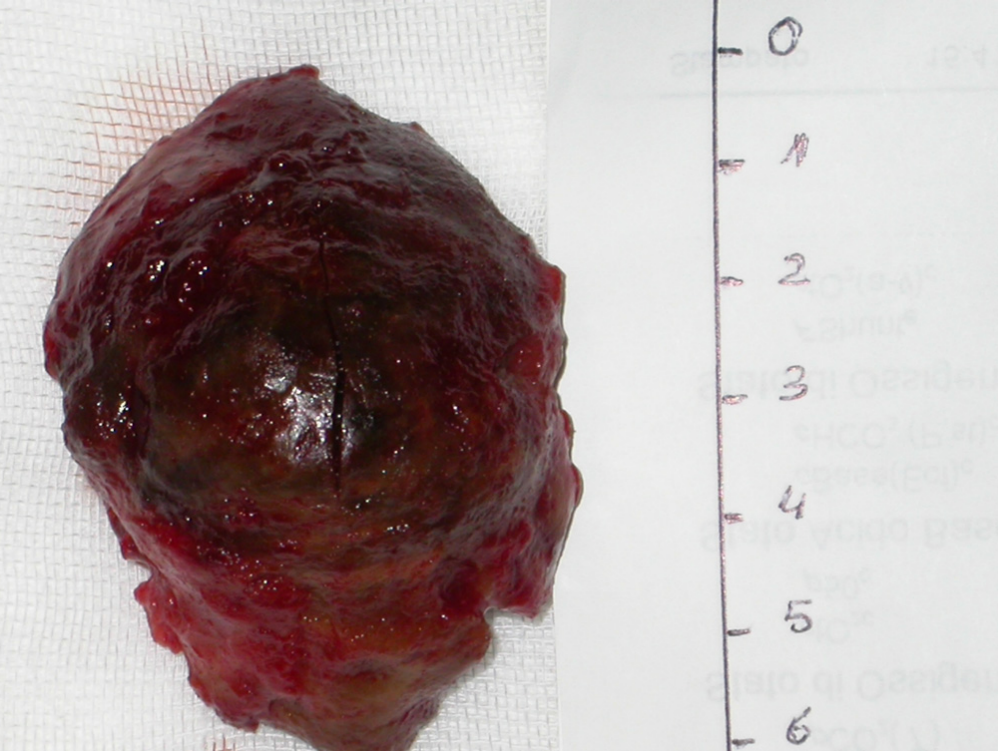
**The thrombus**. The thrombus inside was about 6 cm × 4.5 cm.

At the time of surgery a large true aneurysm of the posterior wall of the left ventricle was found (Fig. 2, 3). The aneurysm consisted of a very thin myocardium layer; inside there was a thrombus about 6 cm × 4.5 cm. (Fig. 4)

**Figure 5:**
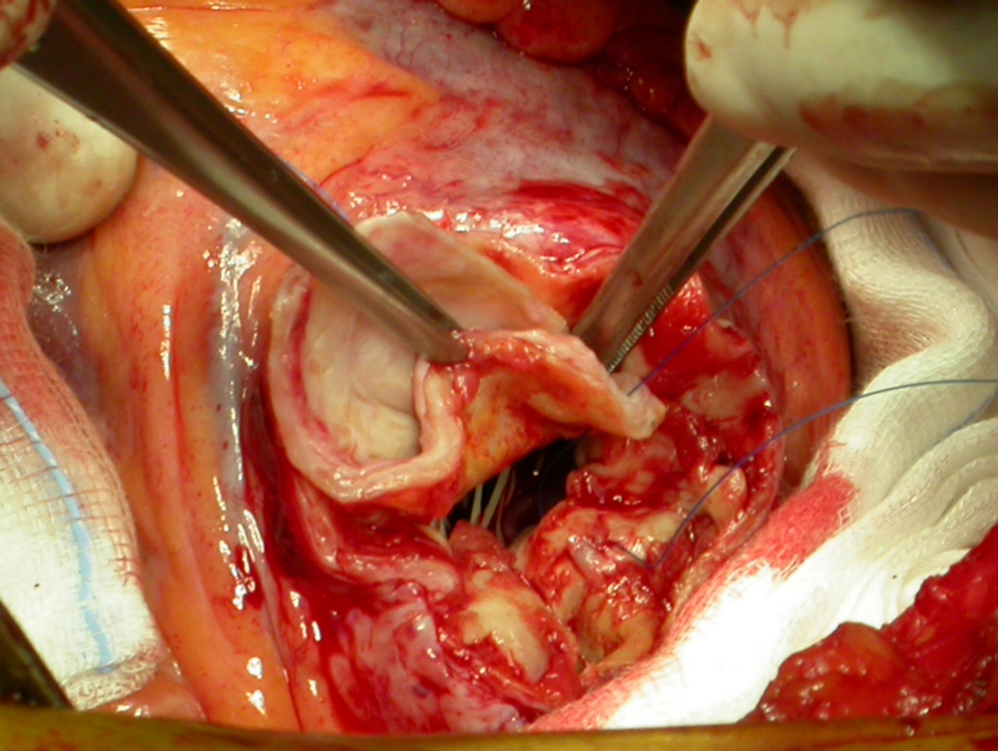
**Dor's repair**. The defect was closed, using the thin autologous aneurysmatic excised myocardium layer as patch.

**Figure 6:**
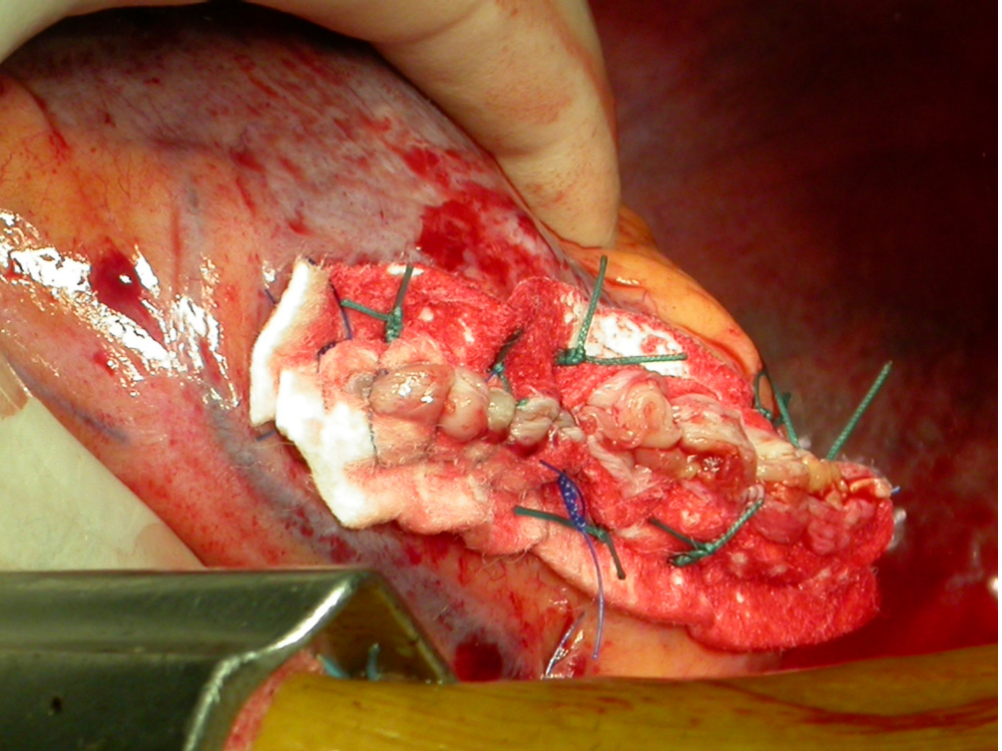
**Teflon pledgets**. Two Teflon pledgets were used to reinforce the ventricle suture on the outside.

Surgery was performed via medial sternotomy. A normothermic cardiopulmonary by-pass (CPB) was carried out with cannulation of the aorta and the right atrium, on a beating heart without cardioplegic arrest and without clamping of the aorta. We performed the Dor repair, as described by Dor et al. [2], after creating a neck of healthy muscle with 2/0 Prolene suture. The defect was closed, using the thin autologous aneurysmatic excised myocardium layer as patch. It was sutured with 3/0 continuous Prolene.(Fig. 5) Two Teflon pledgets were used to reinforce the ventricle suture on the outside.(Fig. 6) The CPB was 78 minutes long. Intra-operative TEE showed no mitral regurgitation (MR) and satisfactory post-surgical remodelling.

The patient was discharged 6 days after surgery without complications. At his 3 month control, the patient was completely restored to a normal life, an echocardiography showed good results in left ventricular remodelling with mild mitral regurgitation and an improved ejection fraction (EF = 55%).

## Discussion

Differentiation between left ventricular aneurysm and pseudo-aneurysm is difficult, yet it is the most important task to carry out when a patient is eligible for surgery.

### Clinical findings

In most cases, patients have no symptoms and an echocardiographic incidental detection of a cavity in the left ventricle wall after a myocardial infarction (MI) is not an answer but rather a diagnostic challenge, as in our case. Otherwise patients can present with chest pain, dyspnea and hypotension [3]. Pericardial friction rub or decreased heart sound, elevation of both left and right side, filling pressure, sinus bradycardia or junctional rhythm, are all signs of pseudo-aneurysm [4]. Persistent ST elevation in the ECG in the area of the infarct is detectable in both aneurysms and pseudo-aneurysms. Distinguishing between these two conditions is very difficult merely on a clinical basis, because many characteristics are common to each.

### Where are the aneurysms located?

The case we present concerns a posterior true aneurysm, but only 3% of aneurysms are posterior or inferior [5]. These data are from an in vivo series, different from an autopsy series that shows an equal distribution of aneurysms respectively in the anterior and posterior location [6]. A clinical preponderance of anterior wall aneurysms can be caused by anterior wall ruptures, generally more fatal than posterior ones, in fact the pericardium in the posterior wall allows the formation of many pseudo-aneurysms. Also posterior infarcts may be lethal because of the involvement of the papillar muscles and consequent severe mitral valve regurgitation, so that an aneurysm cannot easily arise posteriorly. **The more posterior the aneurysm, the more difficult its detection**.

### Echocardiography

This technique plays a prominent role before, during and after surgery. Echocardiography before surgery is useful to differentiate true aneurysm from pseudo-aneurysm: apart from common controls (aortic valve, mitral valve regurgitation, ventricle volumes, atrial volumes, EF), the most important and difficult finding is the detection or not of continuity in the myocardium. Pseudo-aneurysm is a rupture, with discontinuity in the myocardium and loss of blood outside; an aneurysm is a thin myocardium bulging, with outside blood loss. However, when blood clots are present, as in our case, it is difficult to determine if they are inside or outside the myocardium.

A thin or disrupted myocardium moves diskinetically, or the cavity can be non contractile, leading to congestive heart failure, or to dangerous ventricular arrhythmia, and this finding is present in both pathologies. Stagnant flow may lead to thrombosis, or embolic events, thus the mere presence of a thrombus inside the cavity can enable a correct diagnosis. On the other hand, thrombolysis after MI can be dangerous, leading pseudo-aneurysm to a complete rupture. It is also important to differentiate this urgent situation from another one that is pericardial tamponade from thrombolytics in conjunction with PTCA and stent implantation.

Trans-oesophageal Echocardiography (TEE) during intervention, after weaning from cardiopulmonary by-pass, can show immediate results in ventricle restoration, and check mitral continence. An echocardiographic follow-up is the best way to evaluate improvements, especially in EF.

***Cardiac catheterization ***provides information on the degree of coronary artery disease, the degree of mitral regurgitation and the extent of pulmonary hypertension. There are no prospective trials concerning the use of ventriculography in distinguishing aneurysm from pseudo-aneurysm. The two case series published up to now report, as a characteristic of pseudo-aneurysm, a narrow neck connecting the ventricle to the cavity, in which also the contrast liquid also remains for several beats after injection [7,8]. Accurate observation of coronary arteriography can help in making a differential diagnosis because coronary arteries can extend on the aneurysmatic wall in a true aneurysm, while in disrupting the myocardium, a cavity is created by blood and pericardium thus the coronary arteries don't "drape over" the paraventricular chamber of a pseudo-aneurysm [8].

Some authors report that ***CT Angiography ***as a limited role in this kind of diagnosis. The diagnosis can be difficult and the lesions are prone to rupture. Among patients dying of infarction, 17% have been found to have ruptured the heart through the infarcted area. Rupture of the free wall is four to five times more common than septal rupture and is usually immediately fatal. So pseudoaneurym should be recognized s fast as possible and distinguished from the common type of left ventricular aneurysm. Computed tomographic angiography is not available in every centre and his cost is higher than other methods, so should not be considered as first [9].

**Magnetic resonance (MR) **as a role especially for research studies, or for follow-up examinations. Ando S. et al. presenting a serial MR imaging affirm that MR imaging provided the important information for the understanding of the formation process of the pseudo and false pseudo LV aneurysm [10].

Post-infarction LV-remodelling can be characterised by chamber dilatation and abnormal shape leading to systolic and diastolic dysfunction and, in the advanced form, to congestive heart failure. In this condition, the Age-adjusted Survival decreases as shown by Levy et al. in the Framingham Heart Study [11]. Patients in functional class III or IV have a poor 3-year prognosis: in fact only 25% of class NYHA IV survive at 3 years (BMJ 2000).

The clinical situation can help in early or late surgical decision-making, but it is not helpful in distinguishing between the two pathologies. Location is not a useful parameter on which to base diagnosis, (our case was one of the 3% of posterior aneurysms).

Echocardiography is a useful tool but in this pathology, it gives more information to the surgeon than to the cardiologist himself in differential diagnosis. Contrast ventriculography is useful to detect sick coronaries that generate MI. Nearby where the cavity is created, it is important in the evaluation of MR regurgitation but not in distinguishing true aneurysm from pseudo-aneurysm, especially in the case of thrombosis. Surgical approach for true aneurysms has been designed to abort and reverse remodelling, diminish heart failure, and improve survival. Some factors improve surgical outcomes: EF more than 26% before operating, intra-ventricular patch repair, pulmonary artery occlusive pressure of 17 mmHg or less, no need for IAPB, are factors that improve long-term survival after left ventricular remodelling [12].

The aim of surgical therapy in the case of pseudo-aneurysms is relief from ischemia, by performing CABG, diminishing ventricular volume and restoring it to its normal geometry and, when appropriate, bringing further decrease in volume overload by performing mitral valve repair. Yet most of these proposals are common to both, such as excluding akinetic or dyskinetic portions of the ventricle by inserting a patch (we used an autologous myocardial one) at the transitional zone between the contractile and non-contractile myocardium, simply to re-establish ventricular wall continuity. Ventricular volume reduction diminishes wall stress, and thus reduces myocardial oxygen consumption. By excluding the mass of abnormal myocardium, it improves wall compliance, reduces filling pressure, and enhances coronary blood flow. Thus the systolic shortening of fibers is increased in extent and velocity bringing about enhanced contractile performance. Differentiation between aneurysms and pseudo-aneurysm is important in diagnosing and for correct therapy. We have summarized in Table 1 the main differences between aneurysms and pseudo-aneurysms.

**Table 1:** Differences between aneurysms and pseudo-aneurysms.

	**Aneurysms**	**Pseudo-aneurysms**
**Location**	3% posterior	Posterior or inferior

**Echocardiography**		

Anatomy	Thinned myocardium	Ruptures

Contractility	Non contractile	Dyskinesia

**Consequences/Complications**	Congestive heart failure Embolic events Ventricular arrhythmias	

**Therapy**	Medical or Surgical therapy	Surgery

**Surgical risk**	Dubious	Lower than medical therapy

Differentiation between aneurysms and pseudo-aneurysm is important in diagnosing and for correct therapy.

## Consent

A written informed consent was obtained from the patient for publication of this case report and accompanying images. A copy of the written consent is available for review by the Editor-in-Chief of this journal.

## Competing interests

The authors declare that they have no competing interests.

## Authors' contributions

All authors contributed equally to the manuscript, and all authors read and approved the final manuscript.
